# 

**Published:** 1892-08-27

**Authors:** 


### CONTENTS.

PAGE

The State and the Shop - - - - - - - - - 353

Passing Topics.—House Governor or Medical Superintendent?—III. - - - - - - - - - 354

The Doctor's Armchair.—Hypnotism and Hysteria - - - 355

Madmen and their Friends - - - - - - - - - 356

The Attitude of Great Writers towards Disease. XI.—The Literature of Despair - - - - - - - - 357

Everybody's Page - - - - - - - - - 358

Annotations.—Is Cholera Upon Us?—Hills or Sea?—"Cures" for Habitual Drunkenness - - - - - - - - 359

Notes and News - - - - - - - - - 360

Scraps and Gleanings - - - - - - - - - 361

Editor's Letter-Box.—Scarlet Fever - - - - - - 364

The Manchester Crematorium - - - - - - - 364

The Practitioners' Mirror and Retrospect—

Surgery.—The Discussion on the Treatment of Spinal Abscess at the Nottingham Meeting of the British Medical Association 362

Therapeutics.—Asaprol—Phenocoll Hydrochlorate - - - 363

New Drugs, Appliances, and Things Medical.—Diagram of the Mouth, Fauces and Larynx - - - - - - - 364

The Institutional Workshop—

Hospital Administration I.—General Hospitals - - - 365

Asylum Administration.—The Service of Meals in Asylums. By a Medical Superintendent - - - - - - - 366

The Cure of Poverty.—An Account of the North Kensington Friendly Workers among the Poor - - - - - - - 367

The Student of Medicine.—Appointments—Vacancies—Pass Lists.

### EXTRA SUPPLEMENT.—THE NURSING MIRROR.

En Passant.—The Derby and Derbyshire Royal Nursing Institution—Ayr County Hospital—The Brassey Holiday Home — Short Items — Our Christmas Parcels — Brechin Victoria Nursing Association—The Meath Home of Comfort —Preston Royal Infirmary Annual Festivities - - - - cliii

Lectures for Asylum Attendants. By William Harding, M.B. I.—Introductory - - - - - - - cliv

Nursing Homes.—Birmingham General Hospital - - - clv

Royal Cornwall Infirmary, Truro - - - - - - clv

For Reading to the Sick.—Sleeplessness - - - - - clvi

Everybodys' Opinion.—A Hint for a Holiday—Another Hint for a Holiday—A Warning—Massage - - - - - clvi

Appointments—Notes and Queries - - - - - clvii

Wants and Workers - - - - - - - clvii

Some Australian Experiences - - - - - - clvii

Our Holidays - - - - - - - - clviii

Death in our Ranks - - - - - - - clix

"The Flowers of Sleep" - - - - - - - clix

